# Optogenetic Stimulation Using Anion Channelrhodopsin (GtACR1) Facilitates Termination of Reentrant Arrhythmias With Low Light Energy Requirements: A Computational Study

**DOI:** 10.3389/fphys.2021.718622

**Published:** 2021-08-30

**Authors:** Alexander R. Ochs, Thomas V. Karathanos, Natalia A. Trayanova, Patrick M. Boyle

**Affiliations:** ^1^Department of Bioengineering, University of Washington, Seattle, WA, United States; ^2^Department of Biomedical Engineering, Johns Hopkins University, Baltimore, MD, United States; ^3^Alliance for Cardiovascular Diagnostic and Treatment Innovation, Johns Hopkins University, Baltimore, MD, United States; ^4^Institute for Stem Cell and Regenerative Medicine, University of Washington, Seattle, WA, United States; ^5^Center for Cardiovascular Biology, University of Washington, Seattle, WA, United States

**Keywords:** defibrillation, optogenetics, GtACR1, arrhythmia (any), computational simulation and analysis

## Abstract

Optogenetic defibrillation of hearts expressing light-sensitive cation channels (e.g., ChR2) has been proposed as an alternative to conventional electrotherapy. Past modeling work has shown that ChR2 stimulation can depolarize enough myocardium to interrupt arrhythmia, but its efficacy is limited by light attenuation and high energy needs. These shortcomings may be mitigated by using new optogenetic proteins like *Guillardia theta* Anion Channelrhodopsin (GtACR1), which produces a repolarizing outward current upon illumination. Accordingly, we designed a study to assess the feasibility of GtACR1-based optogenetic arrhythmia termination in human hearts. We conducted electrophysiological simulations in MRI-based atrial or ventricular models (*n* = 3 each), with pathological remodeling from atrial fibrillation or ischemic cardiomyopathy, respectively. We simulated light sensitization via viral gene delivery of three different opsins (ChR2, red-shifted ChR2, GtACR1) and uniform endocardial illumination at the appropriate wavelengths (blue, red, or green light, respectively). To analyze consistency of arrhythmia termination, we varied pulse timing (three evenly spaced intervals spanning the reentrant cycle) and intensity (atrial: 0.001–1 mW/mm^2^; ventricular: 0.001–10 mW/mm^2^). In atrial models, GtACR1 stimulation with 0.005 mW/mm^2^ green light consistently terminated reentry; this was 10–100x weaker than the threshold levels for ChR2-mediated defibrillation. In ventricular models, defibrillation was observed in 2/3 models for GtACR1 stimulation at 0.005 mW/mm^2^ (100–200x weaker than ChR2 cases). In the third ventricular model, defibrillation failed in nearly all cases, suggesting that attenuation issues and patient-specific organ/scar geometry may thwart termination in some cases. Across all models, the mechanism of GtACR1-mediated defibrillation was voltage forcing of illuminated tissue toward the modeled channel reversal potential of −40 mV, which made propagation through affected regions impossible. Thus, our findings suggest GtACR1-based optogenetic defibrillation of the human heart may be feasible with ≈2–3 orders of magnitude less energy than ChR2.

## Introduction

Cardiac optogenetics is an emerging field that stems from work involving genetic transduction of light-sensitive ion channels into mammalian neurons (Boyden et al., 2005; Arrenberg et al., 2010). The use of light for current induction in cardiac tissue with precise spatial and temporal precision has led to *in vivo* studies describing selective excitation of specific cell populations (Jia et al., 2011; Addis et al., 2013), control of spiral waves (Burton et al., 2015; Hussaini et al., 2021), and cardiac pace-making (Bruegmann et al., 2010; Ambrosi and Entcheva, 2014; Nussinovitch and Gepstein, 2015a; Vogt et al., 2015) or arrhythmia termination in animal models (Bruegmann et al., 2016; Nyns et al., 2017, 2019; Cheng et al., 2020). *In vitro* applications of optogenetics have yielded all-optical methods for contactless, high-throughput measurement of electrophysiological properties like action potential duration and inter-cellular electric coupling at different spatial scales (Klimas et al., 2016; Boyle et al., 2021). Lastly, *in silico* tools have been created to elucidate mechanisms and test feasibility of optogenetic approaches in larger hearts without the use of preclinical animal models (Nussinovitch et al., 2014; Zaglia et al., 2015; Crocini et al., 2016; Gepstein and Gruber, 2017; Boyle et al., 2018b).

An appealing, long-term translational application of cardiac optogenetics is selectively exciting the heart to terminate arrhythmia. Current standard-of-care treatments for individuals at risk of sudden cardiac death include implantable cardioverter defibrillators (ICDs) and anti-arrhythmic drugs (Siebels and Kuck, 1994; Moss et al., 2002; Poole et al., 2008). While ICDs reduce mortality by eliciting high-energy electrical shocks to defibrillate lethal arrhythmias such as ventricular fibrillation, electrotherapy is also associated with increased mortality, chronic anxiety, and post-traumatic stress disorder (Poole et al., 2008; Pedersen et al., 2011). For individuals with atrial arrhythmias, cardioversion treatments are effective but limited by the in-patient nature of the procedure and the need for anesthesia (Sulke et al., 2007). Optogenetic defibrillation has the potential to circumvent these drawbacks, but prior modeling studies (Bruegmann et al., 2016, 2018; Karathanos et al., 2016; Boyle et al., 2018b) suggest that it would be very difficult to accomplish with current tools, like the channelrhodopsin-2 (ChR2) H134R variant, due to light-attenuating properties of myocardium and high energy requirements.

A potential avenue for moving beyond these limitations is a recently discovered family of opsins called anion channelrhodopsins (ACRs), such as *Guillardia theta* anion channelrhodopsin-1 (GtACR1) (Govorunova et al., 2015). Originally derived from archaea, GtACR1 has desirable cardiac optogenetic characteristics including high single channel conductance, fast response kinetics, specificity in narrow wavelength ranges, and more negative reversal potential values than ChR2 (Govorunova et al., 2015, 2016, 2017). When excited by green illumination, GtACR1 conducts a flow of anions (e.g., Cl^–^), eliciting outward current that accelerates repolarization (Govorunova et al., 2015, 2016, 2017). As such, a new ACR-based paradigm for arrhythmia termination would be distinct from approaches used in past optogenetic defibrillation studies, which have used light-based depolarization to disrupt arrhythmia reentry (Karathanos et al., 2016; Boyle et al., 2018b).

Here, we conduct simulations in patient-derived, biophysically realistic computational models of the diseased atria and ventricles, reconstructed from human late gadolinium enhanced magnetic resonance imaging (LGE-MRI) scans, to investigate the feasibility of GtACR1-based optogenetic defibrillation. Specifically, we aim to determine if uniform endocardial illumination with green light can terminate reentrant arrhythmias in these models with suitable modifications to represent viral GtACR1 expression. Our analysis is designed to reveal (1) the illumination intensity sufficient for GtACR1 to terminate arrhythmia, (2) the mechanisms of defibrillation, and (3) the differences in efficacy between atrial and ventricular models. Simulations with different permutations are used to explore the robustness of light stimulus timing and magnitude. As a basis for comparison, we conduct parallel simulations in the same models expressing blue and red light-sensitive ChR2-H134R variants.

## Materials and Methods

### Computational Modeling of Diseased Atrial and Ventricles

**FIGURE 1:**
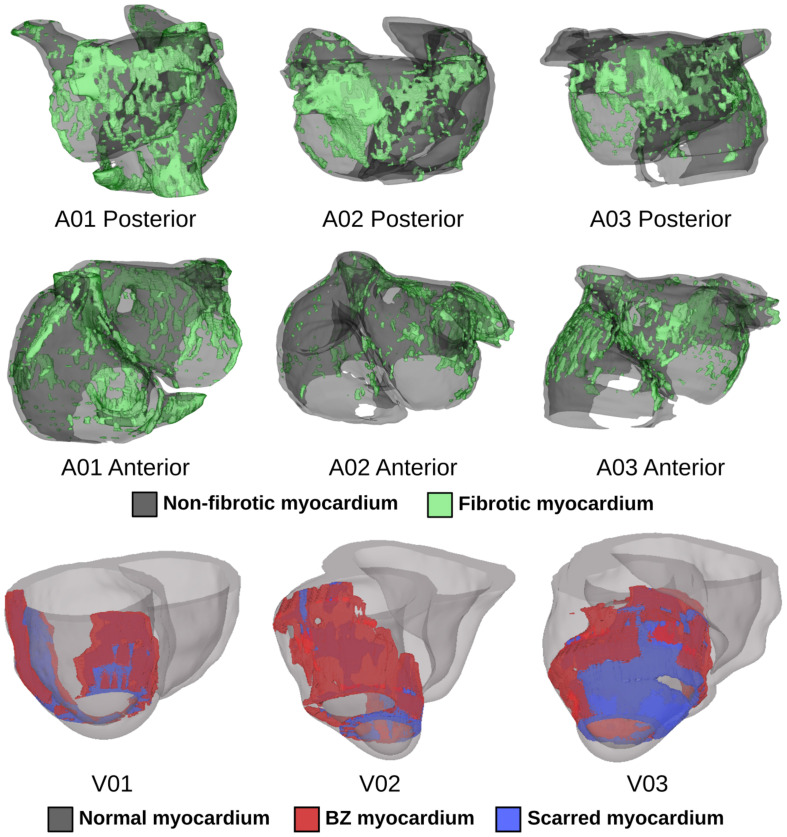
Patient-specific atrial (top two rows) and ventricular (bottom row) models reconstructed from LGE-MRI scans. Spatial distribution of diseased tissue is shown for all cases.

We conducted computational simulations using six patient-specific finite element models (three atrial, three ventricular) reconstructed from LGE-MRI scans (Figure 1). Atrial models were sourced from a cohort of patients with persistent atrial fibrillation (AFib) reconstructed for a prior modeling study (Zahid et al., 2016a); ventricular models came from a different simulation-based study of ischemic cardiomyopathy patients (Arevalo et al., 2016). The approach for simulation of cardiac electrophysiology in such models has been previously validated for their respective applications (Maguire et al., 2003; Ashikaga et al., 2013; Deng et al., 2015); detailed descriptions of atrial (Zahid et al., 2016a,b) and ventricular (Arevalo et al., 2016) simulation methodologies can be found elsewhere (Bruegmann et al., 2016; Karathanos et al., 2016). Briefly, patient-specific geometry and spatial distribution of diseased tissue [fibrotic and non-fibrotic tissue in atrial models (Krummen et al., 2012); normal, scar, and peri-infarct border zone (BZ) tissue in ventricular models (Vadakkumpadan et al., 2010)] were extracted from each patient’s clinical MRI scan (Figure 1). Realistic fiber orientations were introduced in each model. For atrial models, large deformation diffeomorphic metric mapping (LDDMM) was used to transform fiber orientations from an atrial atlas geometry into each patient-specific model. This methodology has been described extensively in prior work (McDowell et al., 2012, 2013, 2015; Zahid et al., 2016a). For ventricular models, we used a rules-based approach that involved solving several Laplacian equations with boundary conditions set on different surfaces of the heart; this has been described and extensively validated by our lab in prior work (Bayer et al., 2012). Human atrial and ventricular myocyte membrane kinetics were represented using the formulations derived by Courtemanche et al. (1998) and ten Tusscher and Panfilov (2006), respectively. As in our earlier studies (Arevalo et al., 2016; Zahid et al., 2016a), average finite element edge length was ∼400 μm in ventricular models and ∼450 μm in atrial models; the temporal discretization was 25 μs in ventricular simulations and 50 μs in atrial simulations. Electrical propagation in cardiac tissue was governed by the monodomain formulation (Vigmond et al., 2003; Plank et al., 2008). As in prior studies using the same cardiac models (Arevalo et al., 2016; Zahid et al., 2016a), ordinary differential equations associated with simulation of action potentials were solved using the Rush-Larsen scheme for ion channel gating variables and forward Euler integration for all other variables; the parabolic partial differential equation was solved with the full (non-lumped) mass matrix using a Crank-Nicholson scheme to improve model stability. All simulations were conducted using the Cardiac Arrhythmia Research Package (CARP) software (Vigmond et al., 2003, 2008; Plank et al., 2008). Patient-specific data used in this study cannot be made publicly available due to data privacy concerns. In the interest of replicability and reproducibility, source files for a complete example of optogenetic stimulation of a publicly available ventricular model (with ChR2, ChR2-RED, or GtACR1) using software tools that are publicly available and free for non-commercial reuse can be found at this link: https://doi.org/10.6084/m9.figshare.14945412. Documentation for this example includes instructions on the use of the openCARP electrophysiology simulator and the meshalyzer visualization software (available via https://opencarp.org/) to run all simulations.

### Mathematical Representation of Light-Induced Current Mediated by GtACR1

We formulated a model of GtACR1 photocurrent kinetics using patch clamp data from GtACR1-expressing neonatal rat ventricular cardiomyocytes as published by Govorunova et al. (2016). Since previous works have suggested structural differences between ACRs and chloride-conducting channelrhodopsins (Govorunova et al., 2017), along with an absence of any reported dark- and light-adapted photocurrent branches in ACRs, we developed a two-state Markov chain model with a conducting [*P*(*O*); light-activated/open] and non-conducting [*P*(*C*); dark/closed] states:

(1) $$\begin{array}{c} P\,\left(O\right)+P\,\left(C\right)=1 \end{array}$$

(2) $$\begin{array}{c} \frac{d\,P\,\left(O\right)}{d\,t}=k_{O\,C} \end{array}$$

(3) $$\begin{array}{c} \frac{d\,P\,\left(C\right)}{d\,t}=k_{C\,O} \end{array}$$

(4) $$\begin{array}{c} k_{O\,C}=\frac{1}{\tau_{o\,f\,f}} \end{array}$$

(5) $$\begin{array}{c} k_{C\,O}=\frac{1}{\tau_{o\,n}}*\frac{5.878134701+\text{ln}\,\left(E_{e}+0.0028\right)}{0.369864-0.1072*\text{ln}\,\left(E_{e}+0.0028\right)} \end{array}$$

where *P*(*O*) is the open-state probability, *P*(*C*) is the closed-state probability, *k*_*OC*_ is the open-to-closed transition rate, *k*_*CO*_ is the closed-to-open transition rate, τ_*off*_ is the inactivation time constant (119 ms), τ_*on*_ is the activation time constant (1110 ms), and *E_e* is the applied irradiance.

**FIGURE 2:**
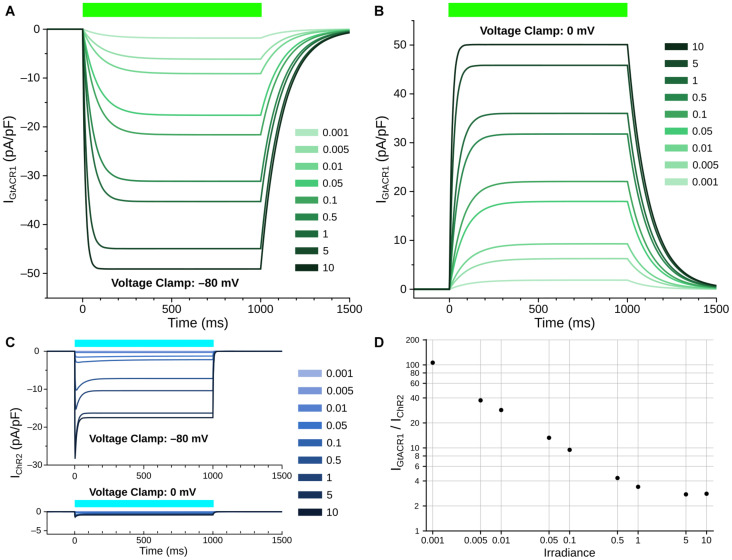
Simulated GtACR1 and ChR2 currents in illumination voltage forcing experiments. **(A,B)** Photocurrent conducted by GtACR1 generated in response to illumination with holding potentials of –80 **(A)** and 0 mV **(B)**. **(C)** Same as A/B but for ChR2. **(D)** Comparison of steady-state I_*GtACR1*_ and I_*ChR2*_ values (plateau currents at t = 500 ms) for different irradiances under voltage clamp condition. In all panels, units of irradiance are mW/mm^2^. Colored bars: intervals of illumination with green (515 nm; 1000 ms) or blue light (488 nm, 1000 ms).

The open-to-closed transition rate (*k*_*OC*_) was calibrated based on inactivation constant of GtACR1, and was fitted via the logarithmic relation of the opening rate to irradiance to ensure that the steady-state currents under illumination matched reported data (Govorunova et al., 2016). The closed-to-open transition rate (*k*_*CO*_) equation varies as a function of irradiance (E_*e*_); *k*_*CO*_ was derived by assuming equilibrium during the photocurrent plateau generated by steady state illumination, then fitting to the open probability *P*(*O*) derived from experimental current traces. To derive maximal GtACR1 channel conductance (*g*_*GtACR1*_), we assumed a membrane capacitance of 100 pF, which is within the physiological range of previously reported NRVM single cell experiments (Guo et al., 1996). The GtACR1 photocurrent model was then characterized at different irradiances under voltage forcing conditions (Figures 2A,B). When a holding potential of –80 mV was used, illumination elicited inward currents, with higher irradiance values leading to larger magnitudes (Figure 2A). With a holding potential of 0 mV, the polarity of the induced photocurrents was reversed (Figure 2B). Using a bisection approach, we found that the best value reconciling current values observed for these clamp levels with experimental data was *g*_*GtACR1*_ = 1.4 mS/cm^2^. We also characterized the ChR2 photocurrent model for comparison to GtACR1 at holding potentials of –80 mV and 0 mV (Figure 2C); GtACR1 currents were several-fold stronger than ChR2 at all irradiances when using –80 mV holding potential (Figure 2D).

Finally, the current density of the channel (*I*_*GtACR1*_) was calculated using Ohm’s law, adjusted for the channel open probability:

(6) $$\begin{array}{c} I_{G\,t\,A\,C\,R\,1}=P\,\left(O\right)*g_{G\,t\,A\,C\,R\,1}*\left(V-E_{G\,t\,A\,C\,R\,1}\right) \end{array}$$

where *E*_*GtACR1*_ is the GtACR1 reversal potential (–40 mV), which we based on prior reports for the reversal potential of chloride ions in cardiac cells under physiologic conditions (Govorunova et al., 2016; Kopton et al., 2018). Notably, the reported reversal potential for GtACR1 in experimental cardiomyocyte preparations was –90 mV (Govorunova et al., 2016); this discrepancy was likely a consequence of bath solution composition.

An implementation of our GtACR1 model compatible with the openCARP framework for cardiac electrophysiology simulations (https://opencarp.org), which is made freely available for non-commercial applications, is provided as supplementary material (see Supplementary Material or https://doi.org/10.6084/m9.figshare.14945412).

### ChR2 Model Variants

The ChR2 photocycles were simulated using an model for ChR2-H134R (Williams et al., 2013), as in previous works (Karathanos et al., 2014, 2016; Bruegmann et al., 2016; Boyle et al., 2018b; Hussaini et al., 2021). Briefly, the ChR2 was modeled as a 4-state Markov chain model with light-gated transitions between two closed (non-conducting) and two open states (permeable to cation flow). The ChR2 model conductance (*g*_*ChR2*_) was originally calculated to be 0.4 mS/cm^2^ based on experiments in HEK-293 cells (Williams et al., 2013), but photocurrents measured from patch clamped cells following viral gene delivery of ChR2 to mouse hearts suggest a lower value (Vogt et al., 2015). Thus, we adjusted our model to fit ∼2.2 pA/pF steady-state currents at 5 mW/mm^2^ illumination (Vogt et al., 2015), which resulted in a conductance of *g*_*ChR2*_ = 0.11 mS/cm^2^. As in prior studies (Bruegmann et al., 2016; Karathanos et al., 2016; Boyle et al., 2018b), we assumed ChR2 stimulation with 488 nm blue light. Since prior modeling studies showed higher defibrillation success rates with red light stimulation (Bruegmann et al., 2016; Karathanos et al., 2016), we also used a theoretical ChR2 model variant with red-shifted absorption (ChR2-RED). This model had the same properties as ChR2 (no modifications to light sensitivity or *g*_*ChR2*_) but peak energy absorption wavelength was adjusted to 669 nm.

### Simulation of Opsin Expression and Light Attenuation

To simulate optogenetic transduction of either the human atria or ventricles via viral gene delivery, we used our previously validated computational modeling framework (Ambrosi et al., 2014; Boyle et al., 2013, 2015a; Bruegmann et al., 2016). Based on mouse experiments studying the effects of gene delivery and expression in cardiomyocytes (Vogt et al., 2015; Bruegmann et al., 2016; Karathanos et al., 2016), opsin expression (ChR2, ChR2-RED, or GtACR1) was incorporated into 58.2% of mesh nodes in a diffuse pattern (i.e., random selection with a uniform distribution), as described previously (Boyle et al., 2013). To facilitate comparison of results across different experimental configurations, we only generated one 58.2% distribution per atrial/ventricular model (i.e., spatial patterns of opsin expression for different opsins were identical).

Light attenuation due to scattering and energy absorption in myocardial tissue was approximated using the steady-state photon diffusion equation (Ripoll et al., 2005; Jacques and Pogue, 2008), as in previous modeling studies (Bishop et al., 2006, 2007; Boyle et al., 2013, 2018a; Ambrosi et al., 2015; Bruegmann et al., 2016; Karathanos et al., 2016). We defined the *a* parameter (values between 0 and 1) using the coefficient for light scattering $\mu_{s}^{\prime }$, coefficient for light absorption μ_*a*_, and the anisotropy factor *g*:

(7) $$\begin{array}{c} a=1-\frac{4}{5}\cdot \frac{\mu_{s}^{\prime }+\mu_{a}}{\mu_{s}^{\prime }\cdot \left(1+g\right)+\mu_{a}} \end{array}$$

We then defined the diffusion coefficient D using the formula (Ripoll et al., 2005):

(8) $$\begin{array}{c} D=\frac{1}{3\,\left(\mu_{s}^{\prime }+\mu_{a}*a\right)} \end{array}$$

We used experimentally derived values found in the literature (Bishop et al., 2006) for blue (μ_*a*_ = 0.52, *D* = 0.183) and red light (μ_*a*_ = 0.1, *D* = 0.34). For green light in cardiac tissue [μ_*a*_ = 0.7, $\mu_{s}^{\prime }$ = 1.42 and*g* = 0.9 (Swartling et al., 2003)] *a* was calculated to be 0.5, leading to a diffusion coefficient of *D* = 0.189. In effect, this means the penetration depth for green light is the shallowest of all the wavelengths used in the study ($\delta =\sqrt{D/\mu_{a}}$ = 519.6 μm vs. 593.2 μm and 1.844 mm for blue and red light, respectively).

**FIGURE 3:**
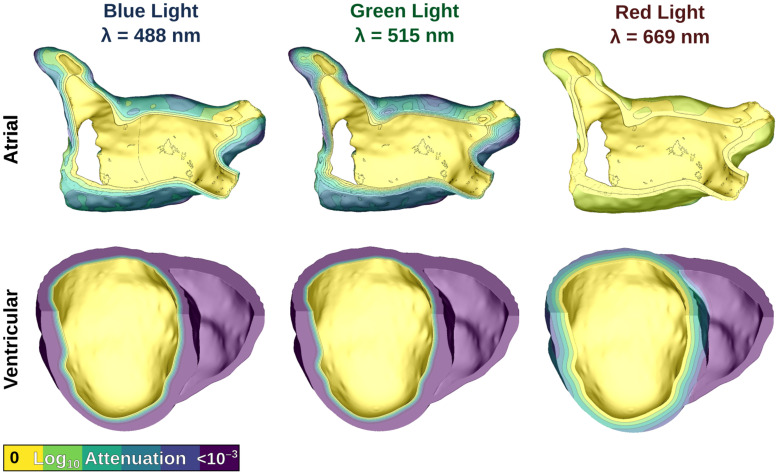
Modeling illumination of atrial (top) and ventricular (bottom) models with light of different wavelengths. Attenuation color scale is shown on a log10 scale. Please note that despite the similarity of the first two panels in the bottom row, these are in fact distinct attenuation patterns for blue and green light.

We simulated uniform illumination of the left atrial (LA) endocardium in atrial models, and the left ventricular (LV) endocardium in ventricular models. Illumination was represented by defining a constant E_*e*_ value on the target surface, then modifying that value by an attenuation factor (derived by solving the photon diffusion equation) (Bishop et al., 2006, 2007; Karathanos et al., 2016; Boyle et al., 2018b) in the myocardial bulk. Figure 3 shows the effects of light attenuation in atrial and ventricular models using the associated wavelengths of each opsin: blue light for ChR2 (488 nm), red light for ChR2-RED (669 nm), and green light for GtACR1 (515 nm).

### Simulation Protocol for Arrhythmia Induction and Optogenetic Defibrillation

Arrhythmias were induced by simulated rapid pacing in both atrial (Zahid et al., 2016a) and ventricular (Arevalo et al., 2016) models, as in prior studies (Karathanos et al., 2016; Bruegmann et al., 2016; Zahid et al., 2016a,b; Boyle et al., 2018b). In atrial models, six electrical pulses of 5 ms duration were paced at coupling interval (CI) = 300 ms, decreasing in 20 ms decrements until CI = 200 ms (6 pulses), followed by six pulses at CI = 200 ms to induce AFib (12 pulses total). In ventricular models, eight pulses of 5 ms duration at CI = 600 ms were initially paced, followed by two pulses at reduced CIs of variable length to induce ventricular tachycardia (VT).

In atrial models, we simulated optogenetic defibrillation attempts with 1000 ms-long LA endocardial illumination pulses at E_*e*_ values varying from 0.001 to 1 mW/mm^2^; these values are consistent with those used in prior experimental studies (Bruegmann et al., 2018; Boyle et al., 2018b; Nyns et al., 2019). In each model, we carried out separate simulation sets assuming expression of ChR2, ChR2-RED, and GtACR1. To span the re-entrant cycle, three light pulse onset times (+0, +70, +140 ms) were used for each E_*e*_/opsin condition. A similar, experimentally consistent (Bruegmann et al., 2010, 2016; Karathanos et al., 2016) protocol was used in ventricular models, with the primary differences being E_*e*_ values (varying from 0.001 to 10 mW/mm^2^), and light onset times (+0, +100, +200 ms) due to longer VT cycle length. In all cases, defibrillation was deemed successful if reentry terminated within 800 ms after the 1000 ms illumination pulse ended; this gave us an adequate timeframe to monitor for resumption of stable reentry (following failed attempts) or indirect successes [as described elsewhere (Boyle et al., 2018b)], wherein spontaneous termination occurs after the end of illumination due to light-induced destabilization.

## Results

### GtACR1 Photocurrent Model Characterization

**FIGURE 4:**
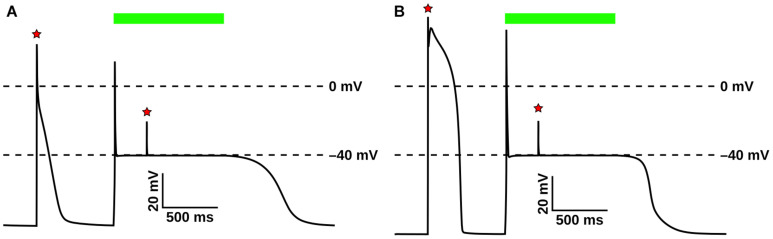
Evaluation of GtACR1 currents upon illumination in single cell simulations. Light-elicited GtACR1 currents suppressed action potential formation in simulated atrial **(A)** or ventricular **(B)** myocyte models. Red stars show timing of electrical stimuli (30 pA/pF) and green bars represent the interval of illumination with green light (515 nm; 1.00 mW/mm^2^; 1000 ms).

The GtACR1 photocurrent model and its response to light were evaluated in atrial (Courtemanche et al., 1998) (Figure 4A) and ventricular (ten Tusscher and Panfilov, 2006) (Figure 4B) myocyte models. In both cell types, following an initial action potential evoked by electrical stimulation (first red star), subsequent light stimulation (green bar) resulted in an abrupt transient depolarization, after which the membrane voltage (V_*m*_) was forced to the GtACR1 reversal potential (–40 mV). This light-induced forcing effect prevented the triggering of subsequent action potentials by electrical stimuli (second red star). After illumination ended, simulated cells repolarized to their resting potentials.

### Optogenetic Defibrillation in Atrial Models

Here, we simulated optogenetic defibrillation attempts in three patient-specific atrial models using LA endocardial illumination at irradiances varying from 0.001 to 1 mW/mm^2^. As summarized in Table 1, GtACR1-based termination was reliable (i.e., reentry activity terminated for all three light onset times tested) for light stimuli as weak as 0.005 mW/mm^2^. Notably, this was 2–3 orders of magnitude lower than the weakest stimuli that reliably defibrillated models expressing ChR2-RED or ChR2 (0.1 and 0.5 mW/mm^2^, respectively).

**TABLE 1:** Defibrillation success rates for ChR2, ChR2-RED, or GtACR1-expressing atrial models for different irradiance values.

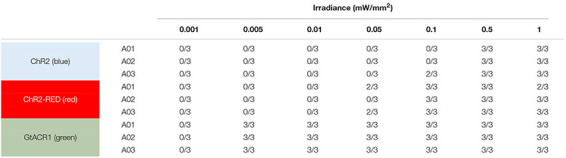

*“n/3” denotes that defibrillation was successful for n of the 3 light onset times tested (i.e., arrhythmia terminated within 800 ms of illumination ending).*

**FIGURE 5:**
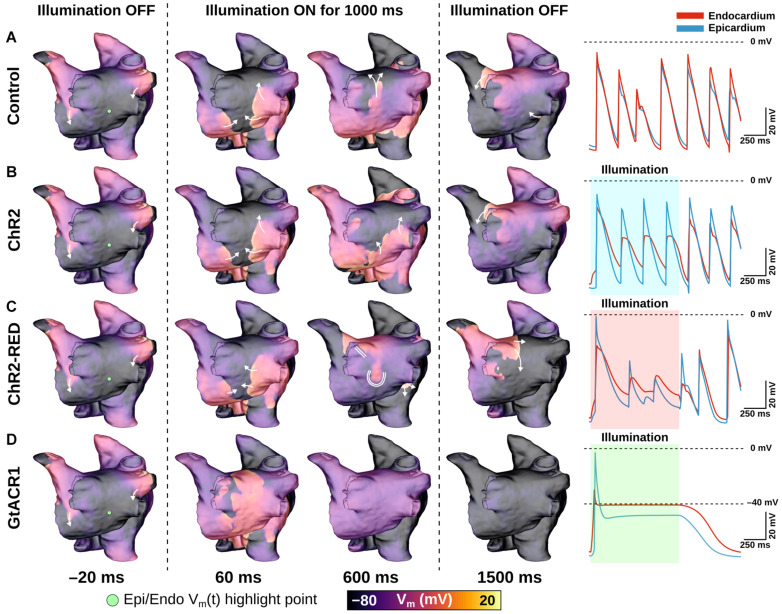
LA defibrillation attempts with E_*e*_ = 0.05 mW/mm^2^ in model A01 for all three opsins. The control case **(A)** is compared to endocardial illumination of ChR2- **(B)**, ChR2-RED- **(C)**, or GtACR1- **(D)** expressing models. Arrows indicate wavefront propagation directions. Double lines show sites of conduction block. Representative V_*m*_(t) traces are shown from endocardial (red line) and epicardial (blue line) nodes at the same point on the LA posterior wall (green dot); shaded regions in rows **(B–D)** indicate illumination intervals. All illumination begins at t = 0 ms and lasts 1000 ms.

The effects of very weak (0.05 mW/mm^2^) light stimuli in atrial models with expression of different opsins are illustrated in Figure 5 and Supplementary Movie 1. In the absence of light stimulation (i.e., control case), reentry persisted; action potential timing and morphology were similar between epicardial and endocardial surfaces (Figure 5A, right-most column). Attempted optogenetic defibrillation in the ChR2-expressing model did not disrupt reentrant activity transmurally (Figure 5B); endocardial action potentials were blunted, but remained temporally synchronized with epicardial excitations, which were largely unaffected by optogenetic stimulation. Stimulation of ChR2-RED had a more prominent effect on the transmural spatiotemporal excitation sequence due to deeper penetration of red light (∼3x exponential decay constant of blue light; Figure 5C); some isolated instances of conduction block were observed (e.g., double lines in 600 ms panel), but arrhythmia did not terminate. In contrast, illumination in the GtACR1-expressing atrial models imposed a voltage forcing effect throughout the LA (Figure 5D, 600 ms), leading to rapid extinguishing of reentrant drivers. Notably, this light-induced forcing effect was non-uniform, with less depolarized plateau voltage at the epicardial surface (spatial gradient: 2.8 mV/mm) due to transmural light attenuation.

**FIGURE 6:**
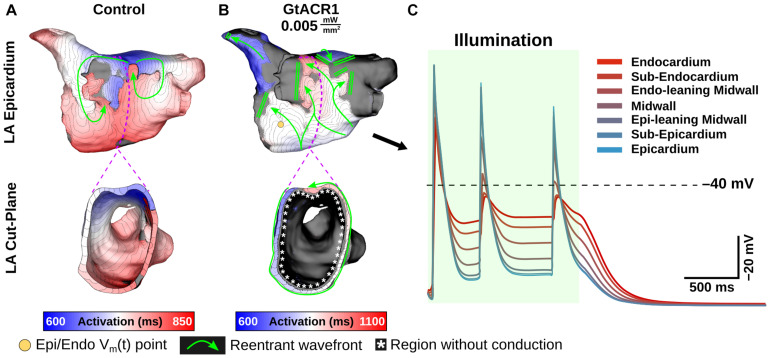
Defibrillation with very weak light stimuli succeeds in GtACR1-expressing models despite persistent sub-epicardial conduction. **(A,B)** Activation maps for no light control **(A)**, and E_*e*_ = 0.005 mW/mm^2^ stimulus applied to GtACR1-expressing model **(B)**. Dashed lines (top) show cut plane locations for cross-sections (bottom). Arrows indicate wavefront propagation directions. Double lines show sites of conduction block. Asterisks highlight conduction block region on endocardium. **(C)** V_*m*_(t) traces for nodes spanning from LA endocardium to epicardium (golden dot) in the GtACR1 condition are provided. Illumination (green shaded region) begins at t = 0 ms and lasts 1000 ms.

At extremely weak irradiances (i.e., 0.005 mW/mm^2^) in GtACR1-expressing atria, light that reached the epicardium was too weak to induce optogenetic voltage forcing. Despite this, defibrillation succeeded in all cases (9/9). To illustrate how this was possible, Figure 6 presents side-by-side activation maps for a GtACR1 defibrillation attempt (E_*e*_ = 0.005 mW/mm^2^) and its corresponding control case (Supplementary Movie 2). In the absence of light stimulus, a reentrant driver in the inferolateral LA propagates unabated (Figure 6A); in contrast, GtACR1 activity elicited by weak illumination of the endocardium disrupted reentry and ultimately terminated the arrhythmia (Figure 6B). Examination of transmural voltage traces in the latter case (Figure 6C) showed that propagating wavefronts in sub-epicardial LA tissue created transient depolarizations from the forced level in the sub-endocardium, but arrhythmia extinguished ∼100 ms after the end of illumination due to light-induced perturbation of excitation patterns near the arrhythmia driver.

### Optogenetic Defibrillation in Ventricular Models

**FIGURE 7:**
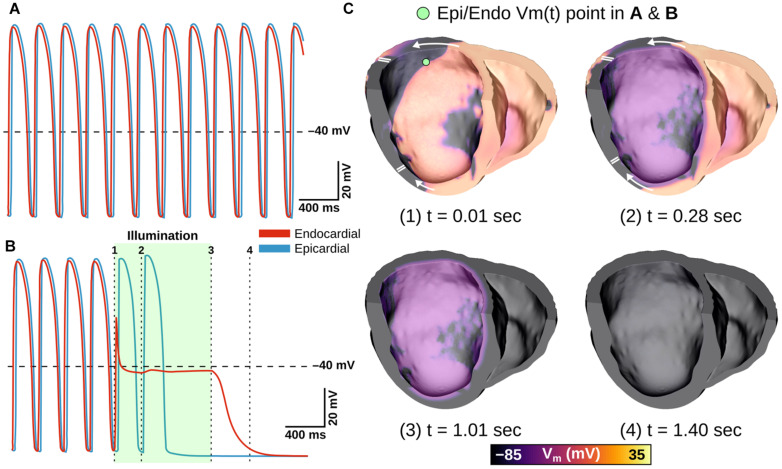
Representative example of LV defibrillation attempt in GtACR1-expressing model V01 with E_*e*_ = 0.5 mW/mm^2^. V_*m*_(t) traces from the LV endocardial surface are provided for the control case **(A)** and GtACR1-expressing model **(B)**. Green shaded region in **(B)** shows illumination interval. Cut plane V_*m*_(t) snapshots of the GtACR1 case **(C)** show conduction block affecting wavefront propagating circumferentially in LV free wall, followed by eventual arrhythmia termination. Arrows indicate wavefront propagation directions. Double lines show sites of conduction block.

Next, we simulated optogenetic defibrillation attempts in three patient-specific ventricular models using LV endocardial illumination at irradiances varying from 0.001 to 10 mW/mm^2^. As summarized in Table 2, GtACR1-based defibrillation was effective with light stimuli as weak as 0.005 mW/mm^2^, which was 2–3 orders of magnitude weaker than for ChR2-RED or ChR2 (0.5 and 1 mW/mm^2^, respectively). A representative example of VT termination (E_*e*_ = 0.5 mW/mm^2^ in GtACR1-expressing model V01) is presented in Figure 7. Without light stimulus, the arrhythmia is sustained (Figure 7A) whereas illumination prevents conduction at the endocardial surface (Figure 7B), resulting in successful GtACR1-mediated defibrillation via light-induced voltage forcing (Figure 7C), like atrial models discussed in the prior section (Supplementary Movie 3).

**TABLE 2:** Defibrillation success rates for ChR2, ChR2-RED, or GtACR1-expressing ventricular models for different irradiance values.

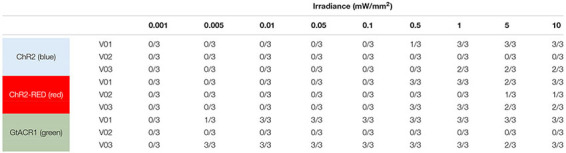

*“n/3” denotes that defibrillation was successful for n of the 3 light onset times tested (i.e., arrhythmia terminated within 800 ms of illumination ending).*

**FIGURE 8:**
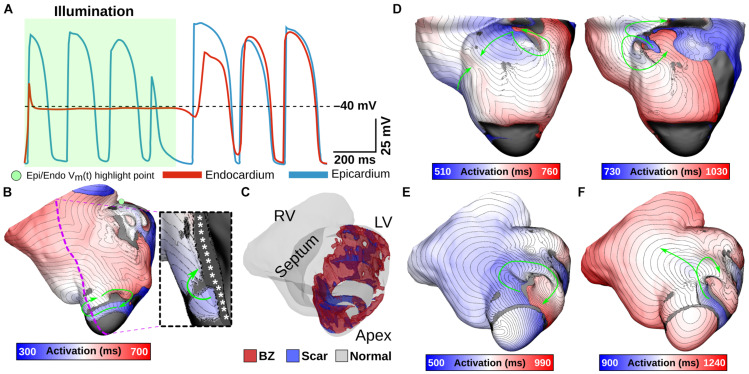
Examining low optogenetic defibrillation success rates in V02. V_*m*_(t) traces **(A)** and an activation map **(B)** showing an example of GtACR1-based defibrillation failure (E_*e*_ = 1 mW/mm^2^). Green shaded region in **(A)** shows illumination interval. Excitation is silenced at the endocardium, but arrhythmia persists at the epicardium (inset) due to a protected VT circuit created by a particular geometry of BZ and scar **(C)**. **(D)** Representative examples to illustrate differences between destabilized reentrant circuits in GtACR1-expressing defibrillation failures (E_*e*_ = 5 mW/mm^2^ and E_*e*_ = 10 mW/mm^2^). **(E,F)** Example of defibrillation success (ChR2-RED, E_*e*_ = 5 mW/mm^2^) showing a normal cycle **(E)** and cycle with conduction block near LV apex **(F)** that leads to termination. Arrows indicate wavefront propagation.

One notable example of inconsistent defibrillation behavior between ventricular models was that defibrillation failed in V02 for nearly all stimuli, regardless of opsin, with success observed in only 2/27 simulations with ChR2-RED. The non-responsiveness of model V02 to optogenetic defibrillation is scrutinized in Figure 8. In a representative GtACR1-expressing defibrillation failure (Figures 8A,B, E_*e*_ = 1 mW/mm^2^), endocardial excitation was suppressed by the optogenetic stimulus (endocardial trace) while the epicardium was unaffected (Figure 8A). Due to light attenuation in the ventricular walls, the proportion of tissue directly depolarized by GtACR1 stimulation was smaller compared to atrial models, so reentrant wavefront conduction continued along a thin layer of epicardium (Figure 8B, inset; Supplementary Movie 4). This was made possible by the presence of a dense ring of scar and BZ near the LV apex (Figure 8C), which created a protected region that was too far from the illuminated endocardium to be affected by the light stimulus and insulated from indirect (electrotonic) effects in areas that *were* optogenetically depolarized. In cases where the reentrant wavefront was dislodged from that area, the arrhythmia driver then relocated to one of many other sites that could sustain a new spiral wave (Figure 8D; GtACR1 expression, examples shown for E_*e*_ = 5 and 10 mW/mm^2^). In the small handful of V02 cases where light-based defibrillation did succeed, termination always occurred several hundred milliseconds after the end of illumination. The apparent mechanism (e.g., Figures 8E,F) was that the dislodged reentrant wavefront serendipitously encountered tissue excited by propagation from another part of the ventricles, resulting in conduction block and subsequent termination.

## Discussion

In this study, we used computational models reconstructed from LGE-MRI scans of diseased human atrial and ventricles to assess the feasibility of reentrant arrhythmia termination via GtACR1-mediated optogenetic stimulation. To achieve this, we developed a 2-state photocurrent model for GtACR1 and evaluated it under realistic organ-scale conditions. In doing so, we showed that GtACR1-mediated optogenetic defibrillation of the atria or ventricles is feasible and more efficacious than a ChR2-based approach, the limitations of which are well known from prior work. Our main findings are: (1) GtACR1-mediated voltage forcing to near the channel Cl^–^ reversal potential of –40 mV consistently terminated arrhythmia in most atrial and ventricular models (3/3 and 2/3, respectively); (2) the threshold irradiance for GtACR1-based atrial defibrillation was extremely low (E_*e*_ = 0.005 mW/mm^2^) in all three cases, corresponding to a ∼10–100x lower energy requirement than ChR2-based optogenetic therapy; and (3) the same very low E_*e*_ threshold was observed in two of three ventricular models.

Optogenetic stimulation is an appealing alternative to electric shocks for defibrillation or cardioversion due to its ability to affect V_*m*_ in the light-sensitized heart alone without triggering excitation and contraction of surrounding skeletal muscle (Ambrosi and Entcheva, 2014; Boyle et al., 2014, 2015b; Karathanos et al., 2016; Crocini et al., 2017). Nearly all past experimental and modeling studies exploring optogenetic defibrillation have used depolarizing opsins such as ChR2-H134R (Entcheva, 2013; Williams et al., 2013; Nussinovitch and Gepstein, 2015a; Karathanos et al., 2016; Boyle et al., 2018a,b), CatCh (Bingen et al., 2014), and ReaChR (Nyns et al., 2017). Opsins that hasten repolarization such as halorhodopsin Cl^–^ pumps (Arrenberg et al., 2010) and bacteriorhodopsin proton pumps (e.g., Arch-3 and ArchT) (Nussinovitch et al., 2014; Nussinovitch and Gepstein, 2015b) have been used previously to silence action potentials in cardiomyocyte monolayers. However, these opsins can only transport a single ion per absorbed photon, resulting in relatively weak photocurrents (Govorunova et al., 2016). Consequently, when ventricular defibrillation via ArchT stimulation was attempted in Langendorff-perfused mouse hearts, termination success was much lower and more variable (∼55%) (Funken et al., 2019) than a comparable study that used ChR2 instead (∼97%) (Bruegmann et al., 2016). Engineered ACRs (Berndt et al., 2014; Wietek et al., 2014) also have limited photocurrent due to the intrinsic pore size of the channels (Guru et al., 2015). In contrast, natural-occurring ACRs (including GtACR1) from cryptophyte algae have higher photocurrents than previous alternatives by an order of magnitude (Govorunova et al., 2015, 2016). As noted elsewhere (Kopton et al., 2018), GtACR1 current depends on the chloride reversal potential (≈–40 mV in cardiomyocytes) and thus *depolarizes* cells at rest while *hastening early repolarization* in cells already undergoing excitation. This differs from the outright silencing of action potentials seen in experimental preparations where the GtACR1 reversal potential was ≈–90 mV due to low [Cl^–^] in the pipette (Govorunova et al., 2016). Nevertheless, our analysis shows that GtACR1-mediated optogenetic defibrillation in large mammalian hearts is theoretically feasible, with suppression of reentrant activity facilitated by forcing large tissue regions toward the reversal potential.

This GtACR1-mediated “optogenetic voltage forcing” mechanism is distinct from depolarization-based routes to termination, as described in prior studies. In the context of constant epicardial illumination of a ChR2-expressing model of infarcted ventricles, Bruegmann et al. (2016) showed the defibrillation mechanism was steady state inactivation of fast Na^+^ channels, resulting in reduced tissue excitability and conduction block; however, in contrast to the present work, the level of sustained depolarization observed in that study (≈–50 mV) was well below the effective ChR2 reversal potential (≈+10 mV) (Williams et al., 2013) due to offsetting effects from other ionic currents. In a different computational study (Karathanos et al., 2016), simulated punctate illumination of the cardiac surfaces by multi-optrode grids in the fibrillating human ventricles under heart failure conditions. In that case, arrhythmia termination was facilitated by creation of new wavefronts via ChR2 stimulation, which eliminated excitable gaps.

Prior studies have identified poor transmural light penetration as a key limiting factor for optogenetic defibrillation of larger hearts, with deeper penetrating red light being highlighted as the most promising potential workaround (Bruegmann et al., 2016; Karathanos et al., 2016). Thus, it is notable that the present study predicts high success rates for optogenetic stimulation based on stimulation of GtACR1-expressing hearts with green light, which has inferior penetration depth compared to both blue and red light. This is a direct result of the lower irradiance threshold for evoking photocurrents in GtACR1 that are large enough to markedly change cardiomyocyte electrophysiology. For example, as shown in Figure 2C using cells clamped to –80 mV, the same current (≈2 pA/pF) elicited by illumination of ChR2 at 0.5 mW/mm^2^ can be achieved by stimulating GtACR1 at 0.001 mW/mm^2^. Thus, for equally bright light stimuli, even though the penetration of green light across the myocardial wall is weaker, the dim illumination of distant regions with attenuated stimuli can produce photocurrents large enough to create a more pronounced electrophysiological effect compared to blue or red light. The recent discovery of red-shifted ACRs from non-algae sources (Govorunova et al., 2020) is also noteworthy in this context, although very slow on/off kinetics (>1 s time constants) make these unsuitable for cardiac applications. Nevertheless, the implication is that opsins even more promising than GtACR1 for optogenetic defibrillation may soon become available. Notably, the modeled virus transfection assumed random dispersion of opsins. This is consistent with prior experimental studies exploring the consequences of long-term ChR2 expression via systemic injection of a viral vector in rodents (Vogt et al., 2015), but it is impossible to know for certain that it would be safe and feasible to achieve this type of distribution in human hearts.

Our computational findings complement a growing body of evidence from experimental work in animal models that light-based cardiac rhythm control is both feasible and efficacious. Initial proof-of-concept work involving optogenetic pacing of Langendorff-perfused transgenic mouse hearts (Bruegmann et al., 2010) and optogenetic modulation of zebrafish heart rate *in vivo* (Arrenberg et al., 2010) prompted speculation that light-based cardioversion and defibrillation might also be feasible (Entcheva, 2013). Subsequent studies demonstrated highly reliable approaches for optogenetic termination of ventricular arrhythmias in explanted mouse hearts expressing ChR2 (Bruegmann et al., 2016) or ReaChR (Nyns et al., 2017), or *in vivo* in open-chest preparations of rats following myocardial infarction (Cheng et al., 2020). Proof of concept has also been shown for light-based defibrillation of atrial arrhythmias in open chest preparations *ex vivo* or *in vivo* (Bruegmann et al., 2018), as well as closed-chest rat models involving automatic detection and termination (Nyns et al., 2019). Prior computational modeling work has suggested that scaling these experiments in larger pre-clinical animal models with contemporary optogenetic tools would be difficult to justify due to constraints imposed by light attenuation in hearts with thicker walls (Bruegmann et al., 2016; Karathanos et al., 2016); the present work suggests that those studies can now be contemplated, thanks to the addition of GtACR1 to the optogenetic armamentarium. However, additional studies may still be needed to verify GtACR1 channel conductance values *in vivo*.

Should delivery of light to beating human hearts prove unfeasible, an interesting potential alternative to the use of GtACR1 could be over-expression of the inward rectifier potassium current I_*K1*_. This would work by a similar mechanism described in this paper, since it would result in depolarization of the resting potential. Moderate upregulation via the I_*K1*_ channel agonist zacopride has previously been shown to reduce triggered arrhythmias in animal models of acute ischemia (Liu et al., 2012; Elnakish et al., 2017; Zhai et al., 2017; Lin et al., 2020). AAV-mediated upregulation of I_*K1*_ ion channels could thus create a similar effect to GtACR1 excitation, although the effects would be at least semi-permanent and extensive safety studies would be needed.

Translation of the cardiac optogenetics for clinical applications remains an attractive goal due to the possibility of pain-free light-based defibrillation replacing electric shock therapy. The present study shows the most convincing evidence to date that arrhythmia in human atria or ventricles could, in theory, be terminated with extremely low-energy light stimuli. Nevertheless, two major hurdles to translation remain, and these parallel two major caveats that must be taken into consideration when interpreting our findings. First, long-term studies are needed to evaluate the safety and durability of opsin expression induced by viral gene delivery to light-sensitize the heart, which has not yet been studied in larger animals. In our study, we simulated distribution of opsin-expressing cells based on reported expression levels in mice one year after AAV9-ChR2 injection (58.2% in a diffuse spatial pattern) (Vogt et al., 2015); a more recent study in rats with hearts light-sensitized by AAV-ChR2 showed defibrillation efficacy one year post-transfection (Li et al., 2021). Although there are no known safety concerns for AAV-based transfection in humans (Wasala et al., 2011; Greenberg et al., 2016), it is not yet known if safe, long-term light-sensitization via ChR2, GtACR1, or any opsin is possible.

Second, even in the context of reduced light energy requirements facilitated by GtACR1, the delivery of sufficient optical energy to the intracardiac milieu in large mammalian hearts remains an unsolved problem. Here, we opted to simulate uniform endocardial illumination, rather than light stimuli focused on specific areas (Boyle et al., 2018b) or delivered by a grid of point sources (Karathanos et al., 2016). We made this choice to facilitate comparison with prior experimental work in animal models, which has used uniform illumination of some kind (Bruegmann et al., 2010, 2016, 2018; Nyns et al., 2017, 2019; Cheng et al., 2020); moreover, whole surface stimulation has been shown to result in lower energy requirements (Quiñonez Uribe et al., 2018). Nevertheless, it would be challenging to use endocardial illumination *in vivo* due to concerns regarding hemodynamic stability. Although it might be possible to implant flexible and biocompatible LED strips (Kim et al., 2010, 2013) along the endocardial surface, it remains unknown whether this type of device would be feasible in practice. An exciting possible alternative is the use of up-converting nanoparticles to facilitate local light release triggered by deeper-penetrating energy like near-infrared light, ultra-sound, and X-rays (Berry et al., 2015; Huang et al., 2016; Boyle et al., 2018a; Entcheva and Kay, 2021). The first proof of concept for optogenetic pacing of rat hearts with this type of technology was recently shown (Rao et al., 2020), but more work will be needed to validate the approach and to determine the most suitable way of representing the relevant physics in our computational models. Finally, our approach to modeling illumination does not account for any inhomogeneities that might arise from uneven light delivery by an LED field or a flexible biocompatible fluorescent membrane (Xu et al., 2014); this simplification was deemed an acceptable tradeoff, since it allowed us to assay feasibility of optogenetic defibrillation in various atrial and ventricular models with distinct organ geometry and functional heterogeneity from disease-related remodeling in a straightforward way.

## Conclusion

We have demonstrated the first computational proof-of-concept for optogenetic defibrillation via stimulation of GtACR1 in biophysically detailed models of diseased human hearts. In all atrial cases and two of three ventricular cases, arrhythmia termination via endocardial light delivery was effective using irradiances as low as 5 μW/mm^2^. The defibrillation mechanism was identified as transmural optogenetic voltage forcing, which was possible because very dim light stimuli can produce large photocurrents in GtACR1-expressing myocytes, thereby mitigating the limitation imposed by light attenuation in cardiac tissue.

## Data Availability Statement

The original contributions presented in the study are included in the article/Supplementary Material, further inquiries can be directed to the corresponding author.

## Author Contributions

TK, NT, and PB conceived and designed the study. AO and TK constructed the computational models, ran the simulations, and analyzed the results. AO, TK, and PB wrote the manuscript. All authors contributed to manuscript revision, read, and approved the submitted version.

## Conflict of Interest

The authors declare that the research was conducted in the absence of any commercial or financial relationships that could be construed as a potential conflict of interest.

## Publisher’s Note

All claims expressed in this article are solely those of the authors and do not necessarily represent those of their affiliated organizations, or those of the publisher, the editors and the reviewers. Any product that may be evaluated in this article, or claim that may be made by its manufacturer, is not guaranteed or endorsed by the publisher.
